# Factors Influencing Nurses’ Opinions on the Implementation of Nursing Advice in Poland

**DOI:** 10.3390/ijerph19137764

**Published:** 2022-06-24

**Authors:** Kinga Harpula, Anna Bartosiewicz

**Affiliations:** 1 Medical College, University of Information Technology and Management, 35-225 Rzeszów, Poland; wilkinga@gmail.com; 2 Healthcare Complex No. 2, Specialist Outpatient Clinic, Diagnostic Center, 35-005 Rzeszów, Poland; 3 Institute of Health Sciences, Medical College of Rzeszów University, 35-959 Rzeszów, Poland

**Keywords:** nurse, nursing advice, nurse prescribing, professional development

## Abstract

In the past five years, nursing practice has changed drastically in Poland. Nurses have received many new competencies in response to the need to provide services to patients. The purpose of the study was to analyze nurses’ opinions on the new rights to provide nursing advice and to identify factors that influence their opinions in this regard. A descriptive cross-sectional study was conducted among 798 nurses who work in various medical facilities. The influence of selected variables on nurses’ opinions on the provision of nursing advice to patients was evaluated using logistic regression. The nurses surveyed had a positive attitude towards new competencies and believed that they were able to independently provide the patient with advice within the scope provided by Polish legislation. Logistic regression showed that the factors that statistically significantly influenced nurses’ opinions on particular types of nursing advice were age (*p* = 0.038), education (*p* = 0.000), and the place of work of the respondents; that is, hospital (*p* = 0.016). More research is needed to demonstrate the effectiveness and quality of the implemented nursing advice and its impact on the functioning of the health system.

## 1. Introduction

Nurses are a key professional group that ensures the functioning and quality of care in medical entities around the world [1]. The recent situation of the COVID-19 pandemic has shown not only the heroism of nurses, but also their skills, thus demonstrating that they are independent and competent specialists [2,3]. Nurses represent more than half of all healthcare sector employees, ensuring the highest level of patient care, which—in an era of deepening staff shortages—further emphasizes their important role [1,4]. Research shows that giving nurses new rights significantly alleviates the effects of the shortage of doctors who, due to the possibility of delegating certain tasks to nurses, can focus on more complicated medical cases. It also enables interdisciplinary cooperation and reduces the professional distance between doctors and nurses [5,6,7]. *Nursing advice* was introduced in Poland in 2020 as a new competency for nurses. The main reason for these changes was the long wait for the doctor’s visit.

The change was introduced in two stages. First, from 1 January 2020, according to the law regulation, *nursing advice* was implemented only for nurses working in outpatient specialist care (surgery, cardiology, diabetology, gynecology, and obstetrics in the case of midwives) [8]. Then, from 1 August 2020, the scope of nursing advice was also extended for nurses working in primary health care [9]. *Nursing advice* is intended for those patients who do not require immediate medical intervention and a doctor’s consultation, but who need a referral, prescription, or ongoing health check [8,9]. Expanding nurses’ competencies has a long history [10], and more and more countries around the world have introduced legal regulations that expand nurses’ powers [11]. The pioneers in this field are the United States (USA) and Canada. In Europe, since 2019, 13 countries have introduced legal regulations extending the rights of nurses (Norway, Cyprus, Estonia, Denmark, Finland, Ireland, France, Netherlands, Poland, Sweden, Spain, United Kingdom and a region of Switzerland) [11,12,13,14]. The benefits of introducing nursing advice are twofold: they give well-qualified nurses greater independence and relieve doctors of activities that do not require their involvement. They also increase the prestige of the profession and the reasons why a nurse is an equal member of the interdisciplinary team and, together with the doctor, provides professional patient care [15,16]. In Poland, of about 260,000 nurses working, 70,000 have the title of specialist in particular fields of medicine [16]. The purpose of the study was to analyze nurses’ opinions on the new rights to provide nursing advice and to identify factors that influence their opinions in this regard.

## 2. Materials and Methods

The study included 798 nurses working in various medical entities of the Podkarpackie Voivodeship in Poland. This is a descriptive cross-sectional study conducted at the end of 2020. Randomly selected medical entities were invited to participate in the study. They received information on the details of the research. Using the algorithm, the sample size was determined. Active nurses who consented to participate in the study were the inclusion criteria. Participation in the survey was voluntary, with the possibility of withdrawing from participation in the survey at any stage and without any consequences. The research tool was a survey questionnaire that contained sociodemographic data of the respondents and questions about nurses’ opinions about the extension of nursing competencies. Respondents responded on a five-point Likert scale from definitely yes to definitely no. The delivery of the paper questionnaires with the enclosed envelope and the possibility of placing the completed forms in sealed envelopes ensured the confidentiality of the responses. The nurses included in the study were representative of all nurses working in the region, and the error threshold was 3%; that is, the post hoc test power analysis was 0.97. The study methodology has been published in detail [17].

### 2.1. Statistical Analysis

Statistical methods used: descriptive statistics: arithmetic mean (M) and standard deviation (SD); Student’s test and analysis of variance (ANOVA). Stepwise logistic regression assessed the influence of selected variables on nurses’ opinions on the provision of nursing advice to patients.

The absolute stability of the questionnaire was established by comparing the results of two measurements performed 6 weeks apart. This assessment was made separately for each question in the survey. The intraclass correlation coefficients obtained ranged from 0.660 to 1.000. The reliability coefficients of the individual questions were also high, ranging from 0.795 to 1.000. All ranges of nursing advice were positively correlated with each other (r range 0.043–0.504) and with the overall result (r range 0.177–0.540). The factor analysis (PCA) using the principal components method, without assuming rotation, distinguished two components that explain a total of 54.11% of the variance of the variable.

A significance level of *p* < 0.05 was assumed.

Calculations were performed with the IBM SPSS program Statistics 20 (IBM, Armonk, NY, USA).

### 2.2. Ethics

The study was approved by the Institutional Bioethics Committee of Rzeszow University (Resolution No. 10/12/2020) and all relevant administrative bodies.

## 3. Results

### Characteristics of the Study Group

A total of 798 nurses were surveyed. The descriptive characteristics of the study group are presented in Table 1.

According to the results obtained, the majority of respondents more or less agreed with the opinion that, due to the extension of nursing rights to the possibility of providing ‘nursing advice’, the functioning of the healthcare system in Poland can be improved. More than half of the respondents expressed the opinion that ‘nursing advice’ would facilitate patients’ access to health services. Additionally, nurses believe that they are capable of properly consulting with the patient without the help of a physician and that they are competent in doing so. Furthermore, the vast majority believe that this will increase the prestige of the nursing profession (Table 2).

Logistic regression considered such predictors as age, work experience, level of education, workplace, and additional qualifications. Table 3 takes into account age and seniority in the regression model. In Table 4, these variables are not taken into account when creating the model, because age and seniority only slightly influenced the opinion of nurses about nursing advice.

The results showed that the selected predictors influenced the opinion of nurses about the scope of nursing advice. The results of odds ratio (OR) > 1 and *p* < 0.05 show which nurses choose a given type of nursing advice more often. The factors that influenced individual types of nursing advice were age (*p* = 0.038), education (*p* = 0.000), and place of work (hospital *p* = 0.016). The OR result <1 shows that the opinion on the prescription of certain medicinal products and medical devices was more often indicated by younger nurses with a master’s degree and working in medical entities other than hospitals. Logistic, progressive step regression allowed us to point out predictors (age; internship; education; workplace: PHC, OSC, long-term care, and hospitals; and additional qualifications: specialization in the field of nursing, qualification courses, specialist courses, and training courses), which statistically significantly influenced nurses’ opinions about the scope of nursing advice (Table 3).

The results demonstrate the key predictors that influence participant opinions on the provision of nursing advice. The prescription of certain medicinal products and medical devices was influenced by education and place of work; people with secondary education (OR = 0.30) and undergraduate education (OR = 0.58) indicated this scope of advice less often than nurses with higher education. Furthermore, nurses who work in the hospital less frequently than their colleagues who work in other places believed that prescribing certain medicinal products and medical devices should include nursing advice (OR = 0.54). Prescriptions as part of continuing treatment ordered earlier by a doctor were indicated less often by nurses with secondary education (OR = 0.29) and working in a hospital (OR = 0.55). Referrals for basic diagnostic tests were less frequently indicated by nurses with secondary education (OR = 0.23), a bachelor’s degree (OR = 0.70), or working in long-term care (OR = 0.56) and in hospitals (OR = 0.52), but more frequently by nurses working in outpatient specialist care (OR = 1.72).

Nurses who completed the training course (expanding and updating knowledge and professional skills) were more often of the opinion that the nurse’s advice should include issuing a referral for basic diagnostic tests (OR = 1.47). Nurses working in OSC (OR = 2.41) and nurses with a specialization in nursing (OR = 1.49) were more likely to provide nursing advice on the interpretation of the results of basic diagnostic tests (OR = 1.49) than nurses working in the hospital (OR = 0.58). The choice of wound treatment method as part of the nursing advice provided was more often indicated by nurses who completed a qualifying course in the field of nursing (OR = 1.54) and a training course (OR = 1.59). This type of nursing advice was reported less frequently by nurses with secondary education (OR = 0.45) and working in PHC (OR = 0.68). The scope of nursing advice related to health promotion and health education was indicated less frequently by nurses working in OSC (OR = 0.35). The possibility of performing preventive examinations as part of nursing advice was indicated more often by nurses working in PHC (OR = 1.54), and less often by nurses with secondary education (OR = 0.31) or undergraduate education (OR = 0.62) (Table 4).

Cronbach’s alpha for the scope of nursing advice was 0.729. All ranges of nursing advice were positively correlated with each other (r range 0.043–0.504) and with the overall result (r range 0.177–0.540). The factor analysis (PCA) using the principal components method, without assuming rotation, distinguished two components that explain a total of 54.11% of the variance of the variable.

## 4. Discussion

In the last five years, the competencies of Polish nurses have significantly expanded. One of their new powers is the ability for nurses of primary care and outpatient specialist care to provide advice to patients, including wound treatment, prescribing prescriptions and referrals for diagnostic tests, patient education, and health promotion [16]. For many years, in Europe, as well as the United States, Australia, and Canada, nurses have greatly broadened the scope of their competencies. As specialists and full members of an interdisciplinary team, they provide high-quality patient care [13,14,15,18]. The education of nurses in Poland, as in other European countries, is based on the Bologna system. This ensures high quality of education and the possibility of working in a learned profession in the countries of the European Union.

Our study showed that the nurses surveyed have a positive attitude towards new competencies. In the opinion of the majority of respondents, this will improve the functioning of the healthcare system, increase patients’ access to health services, and increase the prestige of the profession. Similar benefits resulting from the extension of nurses’ competencies are indicated by other studies in this area [19,20,21]. Furthermore, respondents believe that they are properly prepared and capable of independently providing the patient with advice within the scope provided by Polish legislation [22]. Previous research carried out in Poland indicated that nurses were not too optimistic about extending their competencies and approached new powers with great reserve [23,24]. They assessed their preparation for prescribing as insufficient and saw it as a barrier to taking up new competencies [25]. The fact that the number of nurses’ prescriptions has increased significantly in recent years may prove that the preparation of nurses in this area has improved. According to data from the National Health Fund, the number of nursing consultations is growing consistently year by year. In the case of prescriptions, the situation was similar. In 2016, when the possibility of nurses prescribing was introduced, only 25,000 prescriptions were issued, while in 2021, there were already more than 3 million [16,25,26]. According to Nummimet et al., nurses demonstrate the appropriate level of their competence and are fully prepared to provide specialist advice to patients, including prescribing [27]. Many scientific studies confirm the benefits of extending the scope of nurses’ powers, pointing to the benefits for patients and the entire healthcare system. Benefits also include reduced deaths among patients with chronic diseases and greater patient satisfaction with the care received [28,29,30].

Logistic regression showed that the factors that significantly influenced the opinions of nurses about a given type of nursing advice were age (*p* = 0.038), education (*p* = 0.000), and place of work (hospital *p* = 0.016). In all the cases indicated, the opinion on providing nursing advice, especially on the prescription of specific medical products and devices, was more often shared by young people and by people with a master’s degree than by those with a secondary education in nursing, and more often by people working in medical entities other than hospitals (OR < 1). The list of drugs and medical products that can be issued by a nurse in Poland is regulated by the ordinance of the Minister of Health. Polish legal regulations strictly define the term of a medicinal product as equipment or devices used in healthcare for therapeutic or diagnostic purposes. Among other products are urological catheters, stoma bags, wigs, anti-bedsore mattresses, absorbent products used in incontinence, and diagnostic strips for blood glucose testing [31]. Another study conducted among Polish nurses in this field showed that age was not a statistically significant factor influencing entitlements (*p* = 0.246), in contrast with the number of years of service and the level of education of the respondents. The most positive opinion was given by nurses with work experience between 31–40 years and with a master’s degree [25]. International studies also indicate that taking new powers, e.g., issuing prescriptions, is closely related to having a master’s degree [32,33]. According to the chairman of the Association of Prescribers, which campaigns for and promotes the role of nurse prescribing, the extension of nursing competencies to prescribing has been the most important development in nursing since it became a profession [34]. Research shows that the standard of care provided by nurses is as high as that provided by a doctor, with no significant differences [35,36,37]. Furthermore, it indicates that patients report a high level of satisfaction and trust in nurses who provide advice and prescribing [35,36,37,38]. According to a Cochrane report, in the Netherlands, Ireland, the United States, and the United Kingdom, nurses were as successful in prescribing drugs as doctors [39].

The study limitations were cross-sectional study, territorial limitation—research conducted in the territory of a part of the country—and the short period from the introduction of the nursing advice to the conduction of the study. Furthermore, due to the small number of men who participated in the study and the lack of significant differences in the assessment of nursing advice between women and men, sex was excluded from the regression model. The profession of a nurse in Poland is very feminized. According to the report of the Supreme Chamber of Nurses, 261,000 nurses are currently employed in Poland, of whom only 2% are men [16].

## 5. Conclusions

Extending nursing rights is a common practice in many countries that provides benefits such as improving the quality and accessibility of healthcare for patients. Polish nurses believe that their competencies allow them to take new powers, which will bring a lot of benefits to the health system and patients and increases the prestige of the profession. However, a larger and broader study is needed to discover the impact of nursing advice on the Polish healthcare system. The draft ordinance assumed that nursing advice would replace doctor’s advice from 3% to 7% out of all the advice currently provided by doctors [22].

## Figures and Tables

**Table 1:** Characteristics of the studied group (N = 798).

Independent Variables	Categories	N	%
Age	21–30 years old	238	29.82
	31–40 years old	146	18.3
	41–50 years old	270	33.83
	Over 50 years	144	18.05
Sex	Female	776	97.24
	Male	22	2.76
Work experience as a nurse	1–5 years	223	27.94
	6–15 years	161	20.18
	16–25 years	211	26.44
	Over 25 years	203	25.44
Education	Secondary nursing education	196	24.56
	Bachelor’s degree	311	38.97
	Master’s degree	291	36.47
Additional qualifications	Specialization	245	30.7
	Qualification course	383	47.99
	Specialist course	497	62.32
	Training course	283	35.46
	Other forms of professional development	52	6.52
	No additional qualification	31	3.88
Place of work	Hospital	325	40.73
	Primary health care (PHC)	286	35.84
	Long-term care	92	11.53
	Outpatient specialist care (OSC)	85	10.65
	Health resorts	37	4.64
	Hospice	36	4.51
	Private sector	17	2.13
	Psychiatric care	12	1.5
Scope of nursing advice	Prescribing medications for the patient	474	59.4
	Prescribing medications previously written by a doctor as part of continuing treatment	416	52.13
	Issuing a referral for diagnostic tests	417	52.26
	Interpretation of the results of basic diagnostic and laboratory tests	300	37.59
	Wound healing	512	64.16
	Health education and health promotion	703	88.1
	Prevention	371	46.49

**Table 2:** Nursing advice in nurses’ opinions.

Statement	Categories	%
The new competence improves the healthcare system	Definitely yes	24.69
	Yes	46.37
	I have no opinion	13.41
	No	13.53
	Definitely no	2.01
The new competence increases patients’ access to health services	Definitely yes	19.80
	Yes	45.99
	I have no opinion	14.04
	No	19.05
	Definitely no	19.80
I do not need to consult with doctor to properly consult the patient	Definitely yes	12.66
	Yes	50.63
	I have no opinion	13.53
	No	15.91
	Definitely no	7.20
I am competent to properly perform a consultation for a patient	Definitely yes	13.16
	Yes	52.38
	I have no opinion	16.29
	No	17.17
	Definitely no	1.0
The new powers strengthen the prestige of the nurse profession	Definitely yes	34.71
	Yes	40.60
	I have no opinion	11.78
	No	10.40
	Definitely no	2.51

**Table 3:** Statistically significant variables influencing nurses’ opinions regarding the scope of nursing advice. Logistic regression—stepwise, progressive.

Scope of Nursing Advice		*p*	OR	95% CI OR	
				Down	Up
Prescribing medications for the patient	Secondary nursing education	0.000	0.30	0.20	0.45
	Bachelor’s degree	0.003	0.58	0.41	0.83
	Hospital	0.000	0.54	0.39	0.74
Prescribing medications previously written by a doctor as part of continuing treatment	Secondary nursing education	0.000	0.30	0.20	0.45
	Hospital	0.001	0.59	0.43	0.81
	Health resorts	0.006	3.59	1.44	8.95
Issuing a referral for diagnostic tests	Secondary nursing education	0.000	0.27	0.17	0.40
	Bachelor’s degree	0.039	0.68	0.48	0.98
	Outpatient specialist care	0.015	1.93	1.13	3.27
	Long-term care	0.036	0.60	0.37	0.97
	Hospital	0.004	0.61	0.43	0.85
	Health resorts	0.000	6.07	2.25	16.35
	Training course	0.007	1.55	1.12	2.12
	Other forms of professional development	0.007	2.67	1.31	5.43
Interpretation of the results of basic diagnostic and laboratory tests	Secondary nursing education	0.000	0.44	0.29	0.68
	Primary health care	0.011	1.52	1.10	2.11
	Outpatient specialist care	0.000	3.09	1.91	4.99
	Health resorts	0.000	4.06	1.97	8.37
	Specialization	0.017	1.51	1.08	2.11
	Other forms of professional development	0.048	1.85	1.01	3.42
Wound healing	Secondary nursing education	0.000	0.42	0.28	0.63
	Bachelor’s degree	0.014	0.63	0.43	0.91
	Hospice	0.017	3.05	1.22	7.62
	Psychiatric care	0.042	0.28	0.08	0.95
	Health resorts	0.001	5.05	2.03	12.61
	Qualification course	0.006	1.59	1.14	2.21
	Training course	0.006	1.61	1.14	2.26
	Other forms of professional development	0.049	2.23	1.00	4.97
Health education and health promotion	Outpatient specialist care	0.000	0.37	0.22	0.65
Prevention	Secondary nursing education	0.000	0.30	0.20	0.45
	Bachelor’s degree	0.000	0.53	0.38	0.74
	Primary health care	0.001	1.72	1.27	2.35
	No additional qualification	0.000	6.78	2.68	17.15

**Table 4:** Factors influencing nurses’ opinion regarding the scope of nursing advice. Logistic regression—stepwise, progressive.

Scope of Nursing Advice			*p*	OR	95% CI OR	
					Down	Up
Prescribing medications for the patient	Level of education	Secondary nursing education	0.000	0.30	0.20	0.45
		Bachelor’s degree	0.003	0.58	0.41	0.83
	Place of work	Hospital	0.000	0.54	0.39	0.74
Prescribing medications previously written by a doctor as part of continuing treatment	Level of education	Secondary nursing education	0.000	0.29	0.20	0.44
	Place of work	Hospital	0.000	0.55	0.40	0.75
Issuing a referral for diagnostic tests	Level of education	Secondary nursing education	0.000	0.23	0.15	0.35
		Bachelor’s degree	0.043	0.70	0.49	0.99
	Place of work	Outpatient specialist care	0.042	1.72	1.02	2.91
		Long-term care	0.015	0.56	0.35	0.89
		Hospital	0.000	0.52	0.37	0.73
	Additional qualification	Training course	0.016	1.47	1.08	2.01
Interpretation of the results of basic diagnostic and laboratory tests	Level of education	Secondary nursing education	0.000	0.37	0.24	0.57
	Place of work	Outpatient specialist care	0.000	2.41	1.48	3.91
		Hospital	0.002	0.58	0.42	0.82
	Additional qualification	Specialization	0.018	1.49	1.07	2.08
Wound healing	Level of education	Secondary nursing education	0.000	0.45	0.30	0.67
	Place of work	Primary health care	0.015	0.68	0.50	0.93
	Additional qualification	Qualification course	0.009	1.54	1.11	2.12
		Training course	0.007	1.59	1.13	2.23
Health education and health promotion	Place of work	Outpatient specialist care	0.000	0.35	0.20	0.61
Prevention	Level of education	Secondary nursing education	0.000	0.31	0.21	0.46
		Bachelor’s degree	0.004	0.62	0.45	0.86
	Place of work	Primary health care	0.005	1.54	1.14	2.09

## Data Availability

The data presented in this study are available on reasonable request from the corresponding author: abartosiewicz@ur.edu.pl.
